# Gender-Specific Potential Inhibitory Role of Ca2^+^/Calmodulin Dependent Protein Kinase Phosphatase (CaMKP) in Pressure-Overloaded Mouse Heart

**DOI:** 10.1371/journal.pone.0090822

**Published:** 2014-03-07

**Authors:** Miresta Prévilon, Mylène Pezet, Laurent Vinet, Jean-Jacques Mercadier, Patricia Rouet-Benzineb

**Affiliations:** 1 Inserm, UMRS-698, Université Paris Diderot, Sorbonne Paris Cité, Paris, France; 2 CEFI-Institut Claude Bernard-IFR02, Paris, France; 3 Inserm, U823, Plateforme de Microscopie Photonique – Cytométrie en Flux, Institut Albert Bonniot Site Santé BP170–38042, Grenoble, France; 4 Department of Cell Physiology and Metabolism, University of Geneva, Medical School, Genève, Switzerland; 5 Université Paris Diderot, Sorbonne Paris Cité, Paris, France; 6 Inserm, UMRS-769, Université Paris-Sud, IFR141, LabEx LERMIT, Châtenay-Malabry, France; 7 AP-HP, Hôpital Bichat, Paris, France; Inserm, France

## Abstract

**Background:**

Ca^2+^/calmodulin-dependent protein kinase phosphatase (CaMKP) has been proposed as a potent regulator of multifunctional Ca^2+^/calmodulin-dependent protein kinases (i.e., CaMKII). The CaMKII-dependent activation of myocyte enhancer factor 2 (MEF2) disrupts interactions between MEF2-histone deacetylases (HDACs), thereby de-repressing downstream gene transcription. Whether CaMKP modulates the CaMKII- MEF2 pathway in the heart is unknown. Here, we investigated the molecular and functional consequences of left ventricular (LV) pressure overload in the mouse of both genders, and in particular we evaluated the expression levels and localization of CaMKP and its association with CaMKII-MEF2 signaling.

**Methodology and Principal Findings:**

Five week-old B6D1/F1 mice of both genders underwent a sham-operation or thoracic aortic constriction (TAC). Thirty days later, TAC was associated with pathological LV hypertrophy characterized by systolic and diastolic dysfunction. Gene expression was assessed by real-time PCR. Fetal gene program re-expression comprised increased RNA levels of brain natriuretic peptide and alpha-skeletal actin. Mouse hearts of both genders expressed both CaMKP transcript and protein. Activation of signalling pathways was studied by Western blot in LV lysates or subcellular fractions (nuclear and cytoplasmic). TAC was associated with increased CaMKP expression in male LVs whereas it tended to be decreased in females. The DNA binding activity of MEF2 was determined by spectrophotometry. CaMKP compartmentalization differed according to gender. In male TAC mice, nuclear CaMKP was associated with inactive CaMKII resulting in less MEF2 activation. In female TAC mice, active CaMKII (phospho-CaMKII) detected in the nuclear fraction, was associated with a strong MEF2 transcription factor-binding activity.

**Conclusions/Significance:**

Gender-specific CaMKP compartmentalization is associated with CaMKII-mediated MEF2 activation in pressure-overloaded hearts. Therefore, CaMKP could be considered as an important novel cellular target for the development of new therapeutic strategies for heart diseases.

## Introduction

In cardiac muscle, numerous studies have implicated intracellular calcium (Ca^2+^) as a critical mediator in several signaling functions including contraction and the activation of gene transcription [1]–[6]. Several hypertrophic signaling pathways are interconnected and converge to the nucleus to activate various transcription factors [*7*]. Alterations in Ca^2+^-handling play a pivotal role in pathological left ventricular (LV) remodeling. Two main Ca^2+^-dependent signaling cascades have been identified; the calcineurin-nuclear factor of activated T-cells (Cn-NFAT) pathway [7]–[9] and the Ca^2+^-calmodulin-dependent kinase-myocyte enhancer factor 2 (CaMKII-MEF2) pathway [10]–[12]. The multifunctional CaMKII signaling molecule is considered to play a major role in the twin pathological phenotypes of heart failure and arrhythmia [13], [14]. A subtle balance between phosphorylation (“switch on”) and dephosphorylation (“switch off”) of CaMKs might be directly involved in these deleterious phenotypes.

Among the protein phophatase family, the Ca^2+^-calmodulin-dependent protein kinase phosphatase (CaMKP) belongs to the PPM family of serine/threonine protein phosphatases (EC  = 3.1.3.16) [15], [16]. CaMKP is ubiquitously expressed in all tissues and especially in lung, thymus, brain, spleen, uterus and pancreas [16]. Several reports suggest that CaMKP is involved in apoptotic signaling. On the one hand, the transient expression of either nematode FEM-2 as a homologue of CaMKP participating in sex determination, or rat CaMKP in HeLa cells results in apoptosis [17]; on the other hand, the knockdown of zebrafish CaMKP leads to severe morphological abnormalities indicating its essential role for early development of the brain in this species [18], [19]. Accordingly, dephosphorylation by CaMKP and regulation of multifunctional CaMKs such as autophosphorylated CaMKII, phosphorylated CaMKI and CaMKIV have been reported [15], [16]; [20]–[22]. CaMKP acts as a negative regulator of CaMKs involving important biological processes [21]–[23]. Among many CaMK-regulated transcription factors, myocyte enhancer factor 2 (MEF2) activation triggers physiological and pathological cardiac hypertrophic responses [24].

Nothing is known about the biological role of CaMKP in the heart, which prompted us to investigate whether CaMKP might be involved in CaMKII-mediated MEF2 signaling following pressure overload (PO) of the mouse LV. In addition, because we observed a gender-dependent activation of CaMKII in pressure-overloaded mouse hearts [25] we also aimed at investigating whether CaMKP could be involved in this phenomenon.

## Materials and Methods

### Plasmid construction and purification for mouse CaMKP recombinant protein

Primers for mouse CaMKP (mCaMKP) used for PCR have been designed according to the following sequences containing restriction enzyme sites indicated with bold characters: mCaMKP/***EcoR*** **I**/sense: 5′-CCT**GAATTC**ATGGCAAGCGGTGCAGCACAGAACAGCAGC-3′ and mCAMKP/***Not*** **I**/antisense: 5′-CGT**GCGGCCGC**TTAGCTTCTCTGTGAGGTAT-3′. PCR were performed for 35 cycles (denaturation at 96°C for 30 s, annealing at 60°C for 30 sec, extension at 72°C for 5 min) using platinium Taq DNA polymerase high fidelity (Invitrogen) and transcribed RNA from mouse brain or heart as template. After gel purification and restriction, the digested amplicons were cloned into *EcoR*I/*Not*I digested pSCodon1 to stabilize the vector or GST gene fusion system (PGEX6-P1 vector, GE Healthcare) and sequenced. Competent BL21 bacteria cells were transformed with plasmid DNA (PGEX6-P1/mCaMKP) and grown in Luria Broth medium at 37°C. The subculture of transformed bacteria was grown until one unit of optical density at 600 nm was reached. In order to induce the synthesis of the fusion protein (GST-mCaMKP), the bacterial culture was treated with 100 mmol/L of IPTG for 2 hours at 37°C. Bacterial cells were harvested by centrifugation (5000 rpm for 5 minutes at 4°C), then sonicated at 2.35 Watts for 15 s, lysozyme (10 mg/ml) and DNase-RNase free (10 u/µl) were added. After 10 cycles of freeze (at −80°C for 20 s)-thawing (at 37°C, for 1 minute), the lysate was centrifuged at 13000 rpm for 10 minutes at 4 °C. The supernatant was loaded onto the pre-equilibrated GST-sepharose column (GE-Healthcare). The column was washed twice with phosphate buffered saline, and then equilibrated with scission enzyme buffer. Scission enzyme was added and incubated overnight at 4°C. The recombinant protein (mCaMKP) was eluted by centrifugation at 3000 rpm for 1 minute at 4°C. The recombinant enzyme was used as positive control for phosphatase activity and for immune-characterization.

### Antibody production

Two rabbit polyclonal affinity purified antibodies against the N or C extremity of CaMKP were custom made by Eurogentec. The design of antigenic peptides corresponded to the N-terminus (aa1–14) and the C-terminus (aa 437–452) of CaMKP. These purified peptides were used for immunization of rabbits and to perform the affinity purification of immune serum. The antibodies obtained were used for Western blotting.

### Transverse aortic constriction mouse model (TAC)

B6D2/F1 mice (females and males) were purchased from Janvier (Lyon, France). They were housed in a specific pathogen-free facility and handled according to European Union directives (86/609/EEC) on the care and use of laboratory animals. The review and approval of the study was obtained by the local Animal Ethics Committee (Comité d'éthique Bichat-Debré, Paris-Nord, France; N: B 751803). Mice of both genders (5–7 weeks of age, weighing 18–22 g) underwent TAC without thoracotomy as previously described [26], [27]. TAC was calibrated with a 27-gauge needle placed alongside the aortic arch. Sham-operated (Sham) mice underwent the same surgery, except for the aortic constriction [28]. The control of the success of the TAC procedure has been previously detailed [25]. All animals were randomly divided into groups that were killed by lethal pentobarbital injection (50 mg/kg: intra peritoneal) 30 days after surgery. Body weight (BW) and tibia length (TL) were recorded. After cardioectomy, the cardiac chambers (atria, left (LV) and right (RV) ventricles) were separated and weighed, then quickly frozen in liquid nitrogen and stored at −80°C until use.

### Echocardiography

Transthoracic echocardiography was performed after light isoflurane anesthesia (0.75% to 1.00% in oxygen) and under spontaneous ventilation with a Toshiba Powervision 6000 device, (SSA 370A) equipped with an 8–14 MHz linear and a 6–10 MHz transducer. Echocardiographic measurements were performed as previously described [25]. Transverse aortic velocity flow across the banding (stenotic jet velocity) was measured using a continuous Doppler acquisition mode.

### Gene expression analysis

LV samples were pulverized in liquid nitrogen and total RNA was extracted using TRIzol (Invitrogen). RNA (2 µg) was reverse-transcribed using oligo(dT)_18_ (Invitrogen) and SupercriptII RNAse H-Reverse transcriptase (Invitrogen). To characterize LV molecular remodeling, the RNA transcript levels of various hypertrophic markers such as alpha-skeletal actin (α-SK) and brain natriuretic peptide (BNP) were determined by real-time PCR (LCfast DNA MasterPlus S.G.S E-20, Roche) as were those of glyceraldehyde phosphate dehydrogenase (GAPDH). All primer sequences have been previously reported [28]. Design of sense and anti-sense primers for mouse CaMKP was performed using the previously published cDNA sequence for CaMKP (Genbank, accession number: AK164964). The CaMKP primer sequences are the following: sense: 5′-GCTTCTTCAACTGCCTTTGG-3′ and anti-sense: 5′-CCATGACCATCAAACACAGC-3′. Reactions were carried out in duplicate. Gene expression levels were normalized to GAPDH.

### Western blotting

Frozen cardiac LVs from mice of both genders (sham-operated or TAC) were individually pulverized in liquid nitrogen, to yield a fine powder. The powder was homogenized in lysis buffer (20 mmol/L TRIS-HCl, pH 7.4, 150 mmol/L NaCl, 1% TritonX-100, 1 mmol/L EGTA, 1 mmol/L EDTA, 0.5% sodium deoxycholate) supplemented with a protease inhibitor cocktail (Sigmafast), and the phosphatase inhibitor cocktails 2 (P5726) and 3 (P0044) from Sigma-Aldrich, then solubilized at 4°C for 30 minutes. The LV homogenates were centrifuged at 10,000×*g* at 4°C for 30 minutes. Protein concentrations of the supernatants were determined by the Bradford method, and were in the range of 10–15 µg/µL. Thirty µg proteins of each cardiac LV homogenate were separated by SDS-PAGE, and transferred onto nitrocellulose membranes. Membranes were blocked and blotted with diluted primary antibodies against CaMKP (C- or N-terminus; 1∶5000; custom made by Eurogentec), phospho-CaM kinase II (pThr286; 1∶2000; MA1–047, Affinity BioReagents), CaMKII ((A17); 1∶1000: sc-5393, Santa Cruz, Biotechnology), Oct-1 (1∶2500; MAB5434, Millipore), or histone deacetylase 4 (HDAC4; 1∶1000, #2072, Cell Signaling Technology) and finally incubated with horseradish peroxidase (HRP)-conjugated anti-mouse, anti-goat or anti-rabbit secondary antibodies, as appropriate. Visualization by chemiluminescence was carried out according to the manufacturer's instructions (ECL kit, GE Healthcare). Equal protein loading for LV lysates was assessed by stripping and reprobing blots with an anti-calsequestin antibody (CSQ; 1∶2500, PA1–913, Affinity BioReagents) or an anti-GAPDH antibody (clone 6C5; 1∶2000, Millipore). Quantification of digitized images of immunoblots was done using ImageJ software [http: /rsb.info.nih.gov/nih-image/about.html.]. The intensity of immunoreactive bands was normalized to that of GAPDH or CSQ. Data are expressed as percentages of the respective controls (means ± SEM).

### Histological analysis

Sirius Red staining was used to quantify myocardial fibrosis in mice after surgery. Ten images of 3 sections per animal were taken under the light microscope (Axiostar plus, AxioCAMICc3) at ×20 magnification. The fibrosis score was calculated as (fibrotic area/total image area) ×100.

### LV immunoprecipitated CaMKP (IP-CaMKP) phosphatase activity assay

The frozen cardiac LVs from each mouse experimental group (n = 4 per sham group and n = 8 per TAC group) were individually pulverized in liquid nitrogen using a pestle and mortar, to yield a fine powder. The powder was homogenized in lysis buffer (5 mmol/L TRIS-HCl, pH 7.4, 0.05% TritonX-100, 2 mmol/L EGTA, 2 mmol/L EDTA, 5 mmol/L ascorbic acid, 60 mmol/L dithiothreitrol (DTT), 1mmol/L β-mercaptoethanol) complemented with a cocktail of protease inhibitors (Sigmafast) then solubilized at 4°C for 30 minutes. The LV homogenates were centrifuged at 20,000×*g* at 4°C for 30 minutes. Protein concentrations of the supernatants were determined by the Bradford method, and were in the range of 9–12 µg/µL. Cardiac LV homogenates (150 µg) were immunoprecipitated on magnetic beads coupled to C-terminus CaMKP antibodies. Phosphatase activity of LV IP–CaMKP was measured either by in-gel phosphatase assay using 0.5 mmol/L 4-methylumbelliferyl phosphate (4-MUP, from Sigma), as described previously [29] or with para-nitrophenyl phosphate (pNPP) as a chromogenic substrate. The assay buffer contained 50 mmol/L Tris-acetate, 10 mmol/L MgCl_2_, 20 mmol/L MnCl_2_, 1 mmol/L DTT, 10% glycerol and 20 mmol/L pNPP. Recombinant CaMKP protein as a standard, or LV IP-CaMKP were added to 150 µl of the assay mixture and incubated at 30°C for 30 minutes. The reaction yielding para-nitrophenol was stopped by the addition of an alkaline solution (250 mmol/L NaOH) which produced an intense yellow soluble product measured at 405 nm. Enzyme activity (EA in µmoles/min/µg) was calculated from the Beer-Lambert law expressed as follows: EA =  (V (µL) × OD_405nm_ (cm^−1^))/(ε x incubation time (minute) × enzyme (µg) where ε is the molar extinction coefficient (M^−1^. cm^−1^). For para-nitrophenol, ε = 1.78×10^4^ M^−1^.cm^−1^, OD_405nm_ (cm^−1^) is the absorbance at 405 nm divided by the light-path length (cm). V is the final assay volume, i.e., 150 µL for 96-well plate assay.

### Nuclear and cytoplasmic extracts from mouse left ventricles

Using the Active Motif's nuclear extract kit, the preparation of nuclear and cytoplasmic extracts from mouse hearts were performed according to the manufacturer's instructions. Briefly, the frozen LVs (50–70 mg) were individually pulverized in liquid nitrogen to yield a fine powder, then homogenized in 200 µL ice-cold hypotonic buffer supplemented with the provided DTT, detergent, and phosphatase inhibitors at 4°C and incubated on ice for 15 minutes. The LV homogenates were centrifuged at 850×*g* and at 4°C for 10 minutes. The LV supernatants corresponding to cytoplasmic fractions were stored at −80°C until use. The nuclear pellets were resuspended in 70 µL of complete lysis buffer by pipetting up and down. The suspensions were incubated on ice on a rocking platform (150 rpm) for 30 minutes, and then centrifuged at 14,000×*g* and 4°C for 1 minutes. LV nuclear supernatants were also stored at −80°C until use. Protein concentrations of the cytoplasmic and nuclear supernatants were determined by the Bradford method.

### Myocyte enhancer factor 2 (MEF2) and NFAT activation in mouse hearts

Multiwell colorimetric assay for active MEF2 (MEF2-TransAm) or NFATC1 (NFATC1-TransAm assays, Active Motif Europe) transcription factor was used to determine MEF2 or NFATC1 DNA-binding activities in the absence or in the presence of a competitor oligonucleotide containing the wild-type MEF2 consensus binding site, according to the manufacturer's instructions. Briefly, a biotinylated double-stranded oligonucleotide containing the consensus-binding site for MEF2 was immobilized on streptavidin plates so that LV nuclear extracts containing the active form of MEF2 specifically binds to the consensus binding site in the oligonucleotide. The bound transcription factor was detected with a specific antibody for phosphorylated MEF2 followed by a secondary horseradish peroxidase-conjugated antibody and chromogenic substrate. The colorimetric product was quantified by spectrophotometry at 450 nm with a reference wavelength of 655 nm. A competition assay of DNA-binding activity was performed in the presence of an excess of oligonucleotide (20 pmol) containing the wild type consensus binding site to prove that the transcription isoform detected bound specifically to the immobilized probe. Reactions were run in triplicate. Activation of MEF2 or NFATC1 was expressed as units of OD_450nm_.

### Statistical analysis

Data are expressed as means ± SEM. To determine the effect of surgery, gender and their interaction on parameters measured in LVs, ANOVA for multiple comparisons was used followed by group-to-group comparison with the Bonferroni correction. A p value of ≤0.05 was considered statistically significant.

## Results

### 1- Characterization of molecular tools generated: Recombinant mouse CaMKP and rabbit polyclonal affinity purified antibodies raised against CaMKP-C or -N terminals

**Figure 1:**
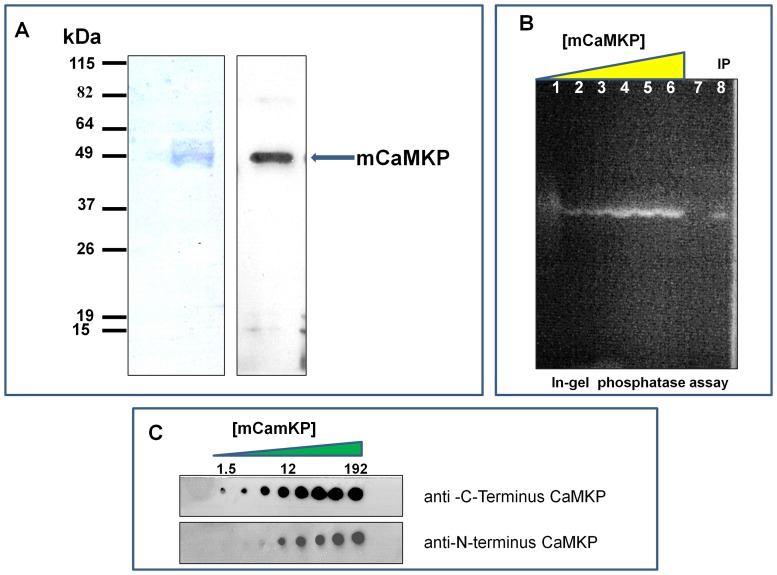
Quality control testing of recombinant mouse CaMKP (mCaMKP). (**A**) Purification of recombinant mCaMKP. Left panel: SDS-PAGE of GST-affinity chromatography-purified mCaMKP stained with Coomassie brilliant blue appearing as a protein band with apparent molecular weight of 49 kDa; right panel: immunoreactivity of purified mCaMKP detected by Western blotting; (**B**) In-gel Phosphatase activity of varying amounts of purified mCaMKP (lanes1–3: 4.5 µg; lanes 4–6: 9 µg; lane 7: none; lane 8: immunoprecipitated mCaMKP (IP)) detected using 4-MUP as a substrate; (**C**) Sensitivity of custom-made antibodies to N- and C-terminals detected by dot blotting using varying amounts of mCaMKP (1.5, 3, 6, 12, 24, 48, 96, 192 ng). Both antibodies recognized mCaMKP terminals, with a slightly stronger signal for the anti-C-terminus antibodies.

The recombinant mCaMKP, purified by GST-affinity chromatography, was observed as a single Coomassie brilliant blue-stained protein band with apparent molecular weight of 49 kDa on SDS-PAGE (Fig. 1A, left panel). The mCaMKP immunoreactivity was confirmed by Western blot (Fig. 1A, right panel). Using 4-MUP as a fluorogenic substrate, phosphatase activity was assayed in agarose gel. Purified CaMKP exhibited a single fluorescent band whose fluorescence intensity increased with the amount of phosphatase loaded (Fig. 1B, lanes 1 to 6). After immunoprecipitation, mCaMKP still exhibited a detectable phosphatase activity in-gel (Fig. 1B, lane 8: IP). Dot blots allowed us to assess the sensitivity of our rabbit affinity-purified polyclonal antibodies raised against the C- or the N-terminal of mCaMKP (Fig. 1C). Both antibodies recognized the recombinant mCaMKP, indicating that both N- and C-terminal domains were present. In this study, recombinant mCaMKP was used as a positive control and all immunodetections were performed with rabbit polyclonal affinity antibodies to the C-terminal CaMKP extremity.

### 2- Cardiac expression of CaMKP

**Figure 2:**
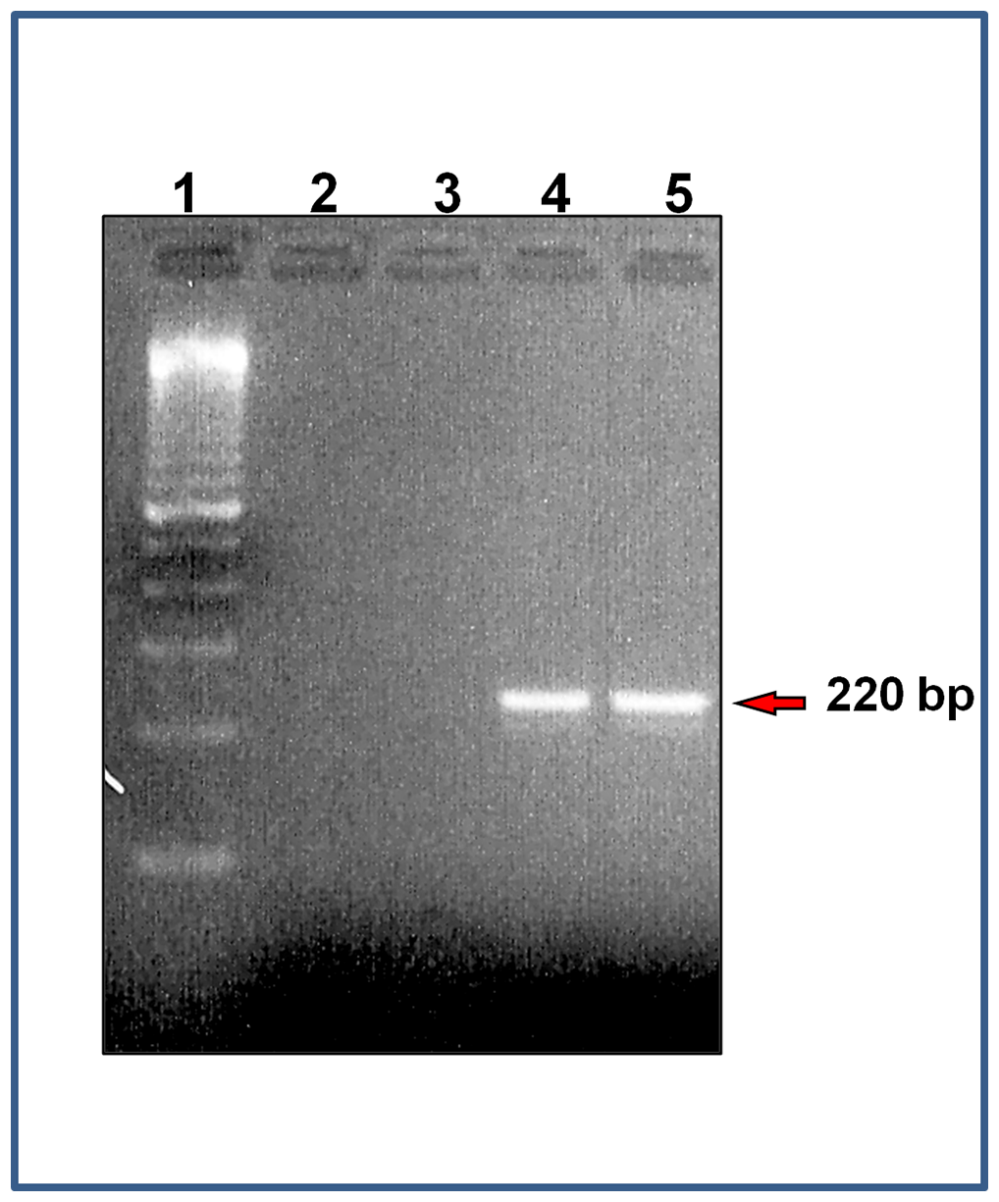
Cardiac expression of CaMKP. (**A**) Total RNA extracted from mouse heart was reverse-transcribed and PCR amplified with CaMKP primers. Lane 1: DNA ladder (100 bp), lane 2: PCR control without DNA template; lane 3: -RT-PCR control (total RNA was not reverse-transcribed); lanes 4–5: a single PCR-amplified product of the exact predicted size (220 bp) for CaMKP was visualized in mouse hearts of both genders (lane 4: male; lane 5: female).

RT-PCR experiments identified an amplified product of the CaMKP gene in LV of both genders as a single amplicon with the expected size of 220 bp (Fig. 2) indicating the expression of the CaMKP transcript in the mouse LV.

### 3- Characterization of TAC mice

**Figure 3:**
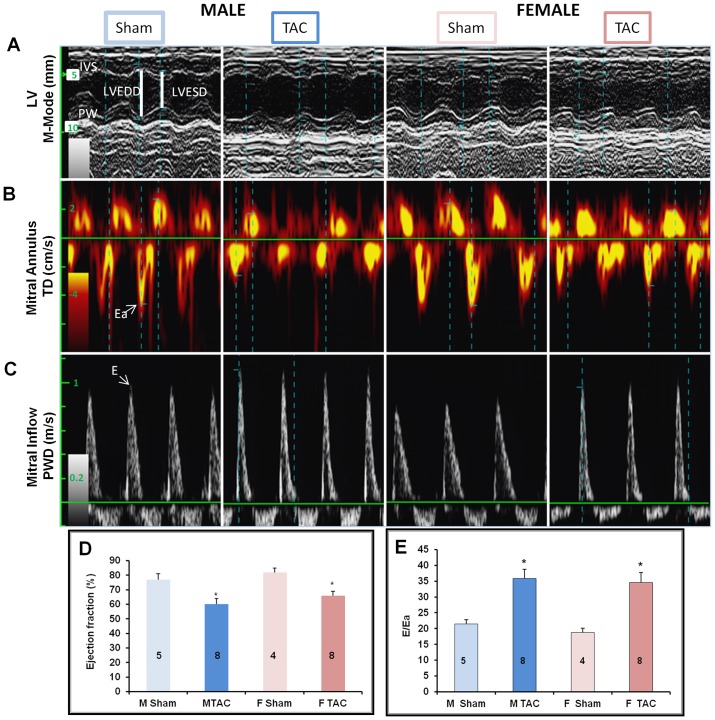
Functional analysis of mouse hearts of both genders. Echocardiographic measurements were performed on mice anaesthetized with isoflurane 30(**A**) Representative echocardiographic images of the left ventricular function. A. Motion-mode of the left ventricle in parasternal long-axis view. IVS, Interventricular septum; PW, posterior wall; LVEDD, Left Ventricle End Diastolic Diameter; LVESD, Left Ventricle End Systolic Diameter. (**B**) Tissue Doppler spectra of the mitral annulus velocity. Ea, early diastolic wave of the mitral annulus (**C**) Pulsed-wave Doppler spectra of the transmitral flow velocity E, peak velocity of the early diastolic mitral wave. All measurements were gated on the ECG trace. (**D**) Ejection fraction evaluation from M-mode measurements. (E) Filling pressure evaluation of the left ventricle (E/Ea ratio). The ratio E/Ea increased similarly in both genders after TAC, indicating diastolic dysfunction. Mice of both genders showed similar systolic and diastolic dysfunction 30 days after TAC. The number of mice is indicated in each column. **M Sham**: male sham-operated mice; **M TAC**: male TAC mice; **F Sham**: female sham-operated mice; **F TAC**: female TAC mice. *p<0.05, TAC *vs.* sham; ^#^ p<0.05, female *vs.* male.

**Figure 4:**
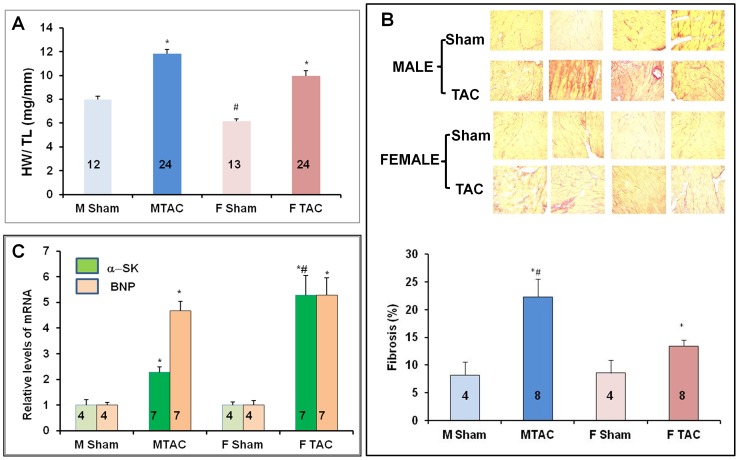
TAC-induced cardiac remodeling. As expected, TAC induced pathological cardiac hypertrophy. (**A**) Heart weight to tibia length (HW/TL) ratios (± SEM) of mice of both genders were determined 30 days post-surgery (**M Sham**: male sham-operated mice; **M TAC**: male TAC mice; **F Sham**: female sham-operated mice; **F TAC**: female TAC mice). (**B)** Top: left ventricular sections stained with Sirius red show interstitial and perivascular fibrosis, (**B**) Bottom: quantification of fibrosis indicated that TAC induced a more severe fibrotic response in male than in female ventricles. (**C**) RNA quantification by real-time PCR of hypertrophic markers: BNP mRNA levels were increased to a similar extent in TAC mice of both genders; alpha-skeletal actin (α-SK) mRNA level was markedly higher in F than in M TAC. The number of mice is indicated in each column. *p<0.05, TAC *vs.* sham; ^#^ p<0.05, female *vs.* male.

Gravimetric and echocardiographic data are reported in Table 1. No difference was observed in stenotic jet velocities indicating a similar degree of aortic constriction in all TAC mice (Table 1). Marked increases in interventricular septum (IVSTD) and posterior wall thickness (PWTD) at end diastole were found in female failing hearts (Table 1). Figure 3A illustrates representative M-mode, pulse wave Doppler and tissue Doppler imaging showing typical E and Ea waves in mice of both genders after either sham-operation or TAC. Both genders of B6D2/F1 mice exhibited the same extent of systolic and diastolic dysfunction 30 days after TAC (Fig. 3B–C, Table 1). In basal conditions, gravimetric analysis showed that heart weight/tibia length (HW/TL) was smaller in female than in male mice (Fig. 4A). As expected, HW/TL was increased in TAC mice of both genders compared with their sham-operated controls (Fig. 4A). Both genders developed the same degree of hypertrophy in response to TAC. As revealed by Sirius red staining, interstitial and perivascular fibrosis were present in pressure-overloaded hearts from both male and female mice (Fig. 4B, upper panel). Quantification of fibrosis (Fig. 4B, lower panel) indicated that male TAC mice (M TAC) exhibited a greater level of fibrosis than female TAC mice (F TAC). BNP and α-SK mRNA levels, a hallmark of pathological LV hypertrophy and heart failure, were also markedly increased after TAC (Fig. 4C). α-SK mRNA levels were markedly higher in female than in male mice whereas BNP mRNA levels were similar.

**Table 1:** Gravimetric and echocardiographic data.

Surgery	MALE		FEMALE	
	Sham	TAC	Sham	TAC
**Gravimetry**				
Number of animals	N = 12	N = 24	N = 13	N = 24
BW (g)	29.8±0.7	27.5±	22.0±0.6	21.9±0.4
HW (mg)	143.1±2.7	202.6±4.6*	106.3±2.4	172.6±4.2*
TL (mm)	17.2±0.1	17.1±0.1	17.2±0.1	17.1±.1
**Echocardiography**				
Number of animals	N = 5	N = 8	N = 4	N = 8
BW (g)	25.0±1.1	24.8±0.7	22.8±0.3	21.4 ±.0.8
HR (bpm)	456±21	443±18	423±19	419±24
IVSTD (mm)	0.61±0.01	0.66±0.08	0.57±0.05	0.83±0.08*#
PWTD (mm)	0.59±0.05	0.77±0.09*	0.51±0.03	0.92±0.12*#
LV mass (mg)	99.9±6.1	149.5±20.2	80.8±5.7	166.2±25.4*
LVEDD (mm)	4.5±0.2	5.0±0.3	4.3±0.1	4.5±0.1*
Spw (cm/s)	3.1±0.1	2.6±0.2*	3.1±0.4	2.3±0.2*
Ea (cm/s)	4.6±0.3	3.3±0.3*	4.7±0.3	3.2±0.4*
Aortic outflow (m/s)	1.38±0.1	1.0±0.1*	1.0±0.1	0.8±0.05*
Stenotic jet (m/s)	-	4.3±0.4	-	4.7±0.4
Velocity ratio	-	4.6±0.7	-	5.9±0.5

Sham, sham-operated; TAC, thoracic aortic constriction; BW, body weight; HW, heart weight; TL, tibia length; HR, heart rate; IVSTD, interventricular septum thickness; PWTD, posterior wall thickness; LVEDD, left ventricular end diastolic diameter; LV mass, left ventricle mass; EF, LV ejection fraction; Spw, maximum systolic velocity of posterior wall; E/Ea, peak velocity of early mitral inflow/early diastolic velocity of the mitral annulus; Velocity ratio, stenotic jet velocity/ LV outflow velocity. *P<0.05, TAC *vs* sham; # P<0.05, female *vs* male.

### 4- Effect of pressure overload on cardiac expression of CaMKP

**Figure 5:**
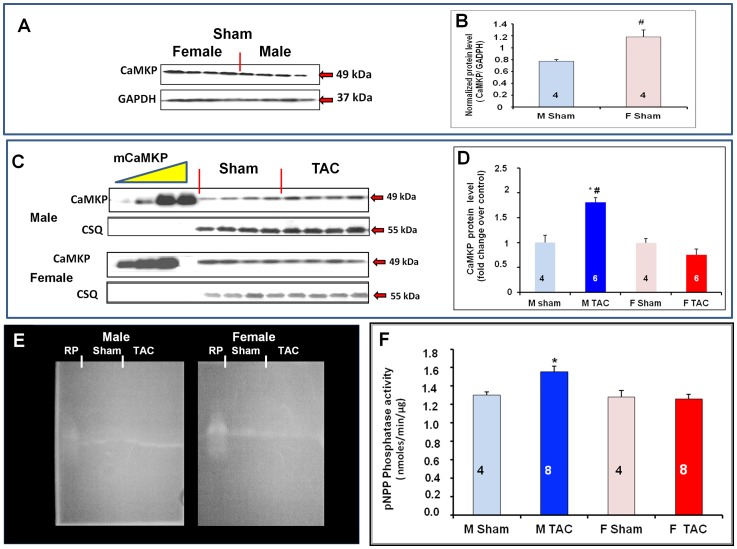
Gender difference in TAC-induced LV CaMKP expression and phosphatase activity. (**A**) Immunoblots from mouse hearts of both genders 30 days after sham-operation. (**B**) Densitometric analysis of Western blot of CaMKP protein levels normalized to GAPDH. At basal level, CaMKP protein level was higher in females (F Sham) than in males (M sham). (**C**) Immunoblots from mouse hearts of both genders 30 days after sham-operation (Sham) or thoracic aortic constriction (TAC). (**5D**) Densitometric analysis of Western blots of CaMKP in TAC mice of both genders. CaMKP protein levels in TAC mice are expressed as fold changes relative to their respective shams. (**E**) In-agarose gel detection of phosphatase activity, using 4-MUP as substrate, of CaMKP immunoprecipitated with anti-CaMKP antibodies from mouse LVs of both genders (left panel: male mice; right panel female mice). RP: CaMKP recombinant protein. (**F**) Phosphatase activity of IP-CaMKP was assayed using para-nitrophenyl phosphate as substrate. *p<0.05, TAC *vs.* sham; ^#^ p<0.05, female *vs.* male.

Gender-dependent differences in CaMKP protein level were seen as shown in the representative immunoblots shown in Figure 5(A–B). In physiological conditions (Fig. 5A), female mice (F Sham) exhibited a significantly higher LV CaMKP protein level than male mice (M Sham) (Fig. 5B). A gender difference in the TAC-associated cardiac CaMKP protein level was also observed: significant up-regulation of CaMKP was seen in male TAC mice (M TAC) whereas female TAC mice exhibited no significant change (Fig. 5C–D).

In order to distinguish the phosphatase activity of CaMKP from that of other phosphatases (i.e., alkaline phosphatases, acid phosphatases, serine/threonine phosphatases and protein tyrosine phosphatases), present in mouse LVs, CaMKP was immunoprecipitated with anti-CaMKP antibodies (LV IP-CaMKP) before measuring its enzymatic activity with either a fluorogenic (4-MUP) or a chromogenic (pNPP) substrate. The in-gel phosphatase assay using 4-MUP suggested a gender difference in TAC-associated CaMKP activity with male TAC mouse hearts exhibiting higher levels of active CaMKP than female TAC hearts (Fig. 5E). This difference was confirmed when phosphatase activity was measured with p-nitrophenyl phosphate (Fig. 5F).

### 5- Gender-dependent CaMKP and CaMKII-mediated MEF2 activation in TAC mice

**Figure 6:**
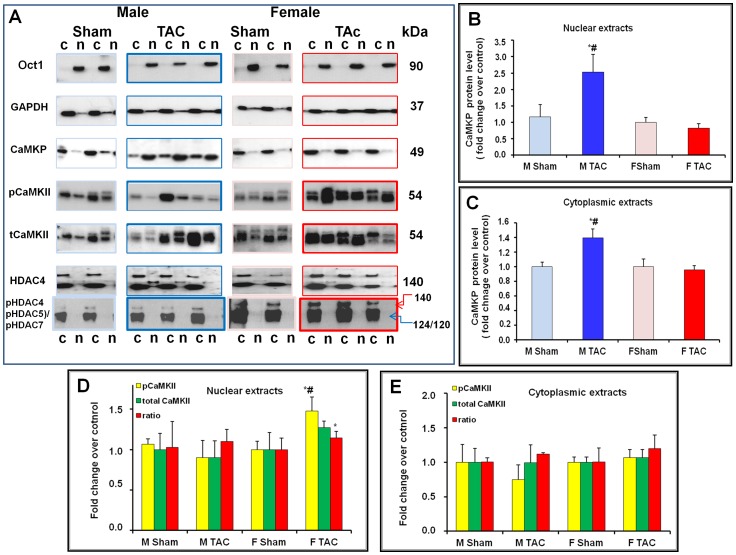
Gender-associated changes in CaMKP and in CaMKII activation in cardiac subcelullar fractions 30 days after TAC. (**A**) Representative immunoblots of Oct1, GAPDH, CaMKP, CaMKII (phospho-Trh286 CamKII and total CaMKII) and HDAC4 (phospho-HDAC4 (Ser246)/HDAC5 (Ser259)/HDAC7 (Ser155) and total HDAC) in cardiac subcellular fractions (nuclear (n) and cytoplasmic (c)) of mouse hearts of both genders 30 days after sham-operation (Sham) or thoracic aortic constriction (TAC). In histograms, blue bars indicate male mice (light blue bar: M Sham; dark blue bar: M TAC), and red bars indicate female mice (light red bar: F Sham; dark red bar: F TAC). (**B – C**) Quantification of CaMKP protein level in LV nuclear (**B**) or cytoplasmic (**C**) extracts reveals a marked gender-dependent up-regulation of CaMKP expression in LV samples after TAC. CaMKP protein levels are increased in both nuclear and cytoplasmic extracts in male mice only. (**D – E**) Phosphorylation status of CaMKII in LV nuclear (**D**) and cytoplasmic (**E**) extracts: phospho-Thr286 CaMKII (yellow bar); total CaMKII (green bar); pCaMKII/tCaMKII ratio (red bar). CaMKP and CaMKII protein levels are expressed as fold increases in TAC mice relative to their respective sham groups. N = 4–8 mice per group. *p<0.05, TAC *vs.* sham; ^#^ p<0.05, female *vs.* male.

**Figure 7:**
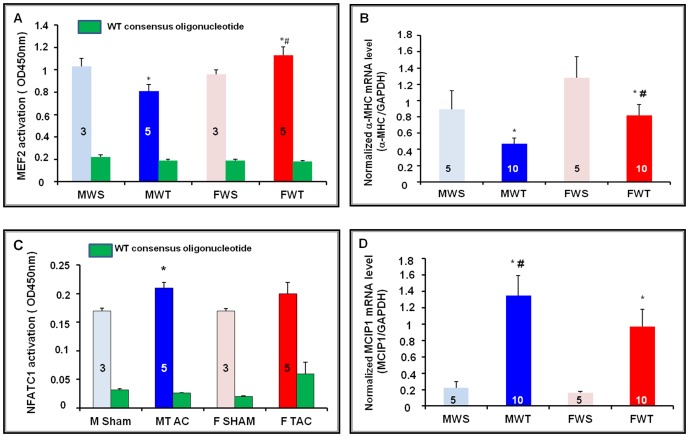
Activation of MEF2 transcription factor in cardiac nuclear extracts. Using the TransAm MEF2 kit, MEF2–DNA binding was quantified by spectrophotometry. (**A**) MEF2-DNA binding activity assayed in the presence of mutated consensus oligonucleotide in 10 µg of nuclear extracts from mouse hearts of both genders 30 days after sham-operation (Sham) or thoracic aortic constriction (TAC), (blue bars for male mice; light blue bar: M Sham; dark blue bar: M TAC; red bars for female mice; light red bar: F Sham; dark red bar: F TAC). The MEF2-DNA binding activity was shifted in presence of the wild-type consensus oligonucleotide at 20 pmol (green bar). (**B**) RNA quantification by real-time PCR of a gen driven by MEF2: alpha myosin heavy chain (α-MHC) mRNA levels were decreased in TAC mice of both genders; but α-MHC mRNA level was markedly higher in females (F TAC) than in males (M TAC). The number of mice is indicated in each column. *p<0.05, TAC *vs.* sham; ^#^ p,0.05, female *vs.* male. (**C**) Using the TransAm NFAT kit, NFAT–DNA binding quantified by spectrophotometry in mouse heart nuclear extracts, and assayed in the presence of the mutated consensus oligonucleotide (color code as for 7A) is significantly increased in M TAC. The NFATC1-DNA binding activity was shifted in presence of the wild-type consensus oligonucleotide at 20 pmol (green bar). (**D**) RNA quantification by real-time PCR of MCIP1 reflected that calcineurin-NFAT pathway activation was higher in male than in female mice after TAC. N = 3–10 mice per group. *p<0.05, TAC *vs.* sham; ^#^ p<0.05, female *vs.* male.

To determine whether CaMKP is expressed within the nucleus and whether this expression varies with TAC, we analyzed by Western blot nuclear (n) and cytoplasmic (c) fractions from mouse hearts of both genders after either sham-operation or TAC (Fig. 6A). GAPDH and Oct1 were used as markers to check for the purity of cytoplasmic and nuclear fractions, respectively (Fig. 6A). As expected, GAPDH was mainly expressed in the cytoplasmic fraction, but a slight expression was also observed in nuclear extracts. Oct1 recognized a 90 kDa protein band in all nuclear extracts only, thus confirming the purity of these extracts. Therefore, we could analyze the distribution of CaMKP in the two heart fractions (Fig. 6A–C). At basal level, similar expression and compartmentalization of CaMKP were observed in mouse hearts of both genders (Fig. 6B–C). In contrast, a gender-specific change in CaMKP expression was seen in TAC mice. Indeed, TAC was associated with increased CaMKP protein levels in both nuclear and cytoplasmic extracts of male mice, a change not observed in female mice. As CaMKP is a serine/threonine phosphatase that can dephosphorylate CaMKII, we also investigated the phosphorylation status of CaMKII in mouse LV fractions (Fig. 6D–E). The endogenous CaMKIIδ was found in both the cytoplasmic and nuclear fractions isolated from mouse LV as two CaMKIIδ immunoreactive bands. TAC was associated with increased levels of active CaMKII (phospho-Thr286) in nuclear extracts of female mice, (Fig. 6D); a feature not observed in cytoplasmic extracts (Fig. 6E). Using anti-phospho-HDAC4 (Ser245)/HDAC5 (Ser259)/HDAC7 (Ser155) rabbit monoclonal antibodies, we investigated the phosphorylation status of HDAC. Representative immunoblots are shown in Figure 6A. Phospho-HDAC 4 (140 kDa) and p-HDAC5/p-HDAC 7 (124/120 kDa) protein levels had accumulated more in female cytoplasmic extracts than in males, whereas HDAC4 protein was mainly present in male nuclear extracts. Taken together, these data suggested that CaMKP could contribute to the CaMKII-mediated MEF activation. Therefore, we investigated the DNA binding activity of MEF2 in mouse cardiac nuclear extracts. Using a colorimetric assay with the antibodies provided, recognizing phosphorylated MEF2 bound to its consensus oligonucleotide only, we observed that active MEF2 was present in mouse nuclear extracts of both genders. MEF2-binding DNA activity was significantly reduced in male nuclear extracts after TAC whereas it was significantly increased in female nuclear extracts (Fig. 7A). The alpha-myosin heavy chain (α-MHC) gene expression is well known to be driven by MEF2Ta. As expected, the expression of α-MHC mRNA was decreased in TAC mice of both genders. However, the decrease was smaller in female than in male TAC mice (Fig. 7B) suggesting that MEF2 transcription was more activated in female than in male mice. In parallel, DNA binding activity of NFAT was significantly increased in nuclear extracts of male TAC mice (Fig. 7C). In addition, differentially increased MCIP1 mRNA levels reflected a gender difference in the activation of the calcineurin/NFAT pathway (Fig. 7D). Cardiac MCIP1 mRNA levels were higher in male than in female TAC mice. Taken together, these data suggested that MEF2 and NFAT activation occurred concomitantly in a gender-specific manner in pressured-overloaded mouse hearts.

## Discussion

The main findings of this study are: 1) the identification of CaMKP and its modulation in the physiological state and pressure-overloaded mouse heart; 2) the observation of a gender-specific compartmentalization of CaMKP upon TAC; 3) the identification of a plausible role of CaMKP, regarding the mechanism that regulates CaMKII-mediated MEF2 activation, by showing its inhibitory effects on CaMKII in a gender-specific manner.

**Figure 8:**
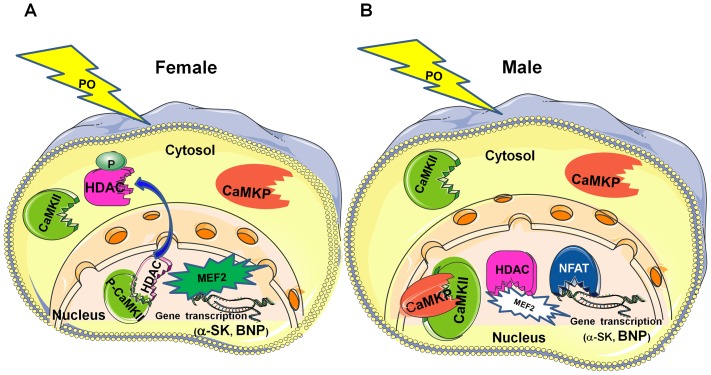
Scenario of TAC-induced gender-dependent CaMKP compartmentalization associated with CaMKII-mediated MEF2 activation in the mouse heart. Diagrams summarizing some CaMKP actions induced by TAC in mouse heart of both genders: (**A**) Following TAC, CaMKP is mainly expressed in the cytoplasm in female LVs and so; the nuclear phospho-CaMKII (active form), pCaMKII, is able to induce HDAC export from the nucleus, relieving, as a consequence, the repression of the hypertrophic myocyte enhancer factor (MEF2). In turn, MEF2 activation results in increased BNP and α-SK gene transcription. (**B**) In males, nuclear CaMKP can dephosphorylate CaMKII. The resulting CaMKII inhibition maintains the repression of MEF2 by HDAC. Decreased MEF2 activation may facilitate the action of other often-associated transcription factors involved in left ventricular remodeling such as NFAT.

TAC-associated insults have been shown to activate a complex set of interwoven signaling pathways that culminate in the nucleus to activate various transcription factors, including myocyte enhancer factor 2 (MEF2), which drive the expression of fetal cardiac and stress response genes. Consistently, in our study, BNP and α-SK mRNA levels were markedly increased in mouse hearts of both genders 30 days post-TAC. TAC was also associated with gender-specific cardiac remodeling, with a more pronounced fibrosis in male mice, in accord with similar observations previously reported [30]–[34]. Using home- and custom-made molecular tools, we showed that mouse heart expressed the serine/threonine phosphatase CaMKP, at both the mRNA and protein levels. Moreover, we demonstrated a gender difference in the link between the expression of CaMKP and the activation of the CaMKII-MEF2 signaling pathway in response to chronic pressure overload (PO). TAC induced the up-regulation of CaMKP protein expression in male mouse hearts. A gender difference in CaMKP compartmentalization was also observed after TAC. Because cardiac subcellular fractionation was performed in the presence of multiple phosphatase inhibitors, it has not been possible so far to determine the phosphatase activity of CaMKP. Neither could the *in situ* CaMKP phosphatase activity be determined. Based on *in vitro* phosphatase activity determined either by in-gel phosphatase activity assay [29] or using the generic phosphatase substrate pNPP in total LV homogenates after immunoprecipitation with antibodies raised against CaMKP, we found that TAC induced a gender-specific alteration in CaMKP activity that was increased significantly in male TAC mice only (Fig. 5). Moreover, we observed that CaMKP protein levels were higher in nuclear than in cytoplasmic extracts in male TAC mice, only, suggesting CaMKP translocation into the nucleus. In addition, the nuclear localization of both CaMKP and CaMKII allowed us to propose a scenario occurring in male mouse hearts upon PO (Fig. 8B). However, whether or not CaMKP translocation is the primary event leading to the negative regulation of CaMKII during chronic PO cannot be answered by our data. Whatever the initial event, our data point out the possibility that CaMKP dephosphorylates CaMKII with, as a consequence, a decrease or even an absence of MEF2 activation, explaining the small α-SK mRNA accumulation in male TAC LVs. Our findings regarding the phosphorylation status of CaMKII and its targets (HDAC4, MEF2) in cardiac nuclear extracts argue for CaMKP regulating the CaMKII-mediated MEF2 activation in a gender-specific manner. Although the weak CaMKP protein level does not prove a causal relationship, we also propose that the presence of p-CaMKII in the nuclear fraction from female TAC mice (Fig. 8A) resulted in the cytoplasmic accumulation of phospho-HDAC4 (Fig. 6A). These observations concerning CaMKII are consistent with previous reports showing that during cardiac adaptation to exercise [35] and in chronic heart failure, CaMKII is typically activated [36], [37], together with the cytoplasmic accumulation of HDAC4 [38], [39]. The MEF2 family transcription factors interact with HDACs and regulate downstream gene expression for several hypertrophic signaling pathways in the heart [35], [40]–[42]. Although knocking out class IIa HADC in the heart appears to be generally detrimental, class IIA HDAC deletion is beneficial in some cases. Female mice lacking HDAC5 or HDAC9 are protected from post-myocardial infarction (MI) remodeling due to enhanced estrogen receptor-mediated transcription of proangiogenic genes in the heart whereas male knockout mice die at a higher frequency post-MI [44]. In addition, mice expressing high cardiac levels of CaMKIIδ develop hypertrophy and dilated cardiomyopathy whereas deletion of CaMKIIδ in the mouse heart results in attenuated pathological cardiac hypertrophy. Indeed, CaMKII signals selectively target HDAC4 by docking to a domain that is uniquely present in HDAC4 and not in other class IIa HDACs [38], [39]. MEF2 only becomes active with the phosphorylation and nuclear export of HDAC4 by CaMKIIδ [41]–[43]. Consistent with this report, despite nuclear export of HDAC4, measurement of the MEF2-DNA binding activity allowed us to establish that MEF2 was phosphorylated. Moreover, an accumulation of α-SK mRNA was also found in female TAC mice, consistent with a previous report involving MEF2 in the regulation of the expression of skeletal actin expression in the heart [45]–[46]. In addition, it was shown recently that the effects of nuclear CaMKIIδB on chromatin structure depend on its kinase activity and are critical for MEF2 transcription from chromatin templates during cardiac hypertrophy [47]. Although CaMKP is also a Ca^2+/^calmodulin-dependent enzyme, as are calcineurin and CaMKII, the precise mechanism(s) by which these enzymes affect Ca^2+^ stores during cardiac hypertrophy, and in subsequent heart failure, are still unclear. Despite the different expression levels and localization of CaMKP and consequent CaMKII-MEF2 activation, mice of both genders developed the same degree of hypertrophy and dysfunction, suggesting that a variety of different concomitant signal transduction pathways lead to a similar degree of LV hypertrophy and dysfunction. In parallel, we also observed a gender difference in calcineurin-NFAT pathway activation. An increased MCIP1 mRNA levels reflecting this activation was accompanied by increased NFAT-DNA-binding activity which in turn instigated an increase in BNP mRNA levels in male mice after TAC. Although gender differences in the activation of CaMKII and calcineurin-NFAT have been previously shown [25], it is still unknown how the nucleus regulates its calcium entry and exit to regulate CaMKP localization and function following TAC.

The important question of how the nucleus gains its autonomy to regulate the difference in CaMKP compartmentalization after TAC is open to debate. Recently, CaMKP-N, a new isoform of CaMKP, was shown to translocate from the nucleus to the cytosol after proteolytic processing [48]. Nuclei are dynamic organelles capable of orchestrating multiple convergent signals such as calcium and/or redox signaling which can also modify structure and function of proteins and impact their compartmentalization. Indeed, reactive oxygen species (ROS) are known to oxidize sulfhydryl (SH) groups of cysteine residues in proteins, which can lead to the formation of disulfide bonds, which in turn can affect the quaternary structure of proteins and alter their function. Only recently, it was shown that the incubation of CaMKP with H_2_O_2_ caused formation of a disulfide bond resulting in inactivation of the enzyme [49]. Many Ca2+-handling proteins have been shown to be targets for ROS-dependent oxidation but the physiological and/or pathophysiological relevance of this remains largely unknown [50]. ROS can also oxidize methionine residues (i.e, methionine 281/282 in the regulatory domain CaMKII), thereby resulting in an activation mode very similar to autophosphorylation at T286 [51]. The role of oxidized CaMKII in the cardiovascular system was reviewed recently in detail [52]. Further complexity is added to such post-translational events which could also occur during the TAC-associated insults. One may also question how gender impacts calcium and/or redox signaling.

### Conclusions

The present work provides novel information regarding the mechanisms that regulate CaMKII-mediated MEF2 activation in pressure-overloaded mouse heart, particularly by revealing the possible inhibitory role of CaMKP on CaMKII in a gender-specific manner. Further work is required to determine the up-stream molecular mechanism of this gender-specific compartmentalization of CaMKP following TAC. Moreover, it is becoming increasingly apparent that this mechanism of CaMKP needs to be evaluated in a gender-selective manner. Further research, aimed at identification of gender differences in cardiomyocyte CaMKP activation/inhibition and the associated signaling processes, may provide a basis for the development of gender-specific cardiac therapies.
